# Premature Death

**Published:** 1878-07

**Authors:** 


### PREMATURE DEATHS.

The suicides in France now average ten a day; the number for the present century, thus far, is over three hundred thousand. Not a day passes in which a suicide may not be directly traced to want of success in life; to the false moralities inculcated by wicked or ignorant writers; to the failure of parents in obtaining a proper influence over their children; to unrestrained appetites and passions; and to the inability of multitudes "to get along in the world" prosperously, for want of thoroughness of preparation for their calling or station in life.

